# Student nurses’ career intentions following placements in general practice through the advanced training practices scheme (ATPS): findings from an online survey

**DOI:** 10.1186/s12909-019-1880-8

**Published:** 2019-12-03

**Authors:** Robin Lewis, Rachel Ibbotson, Shona Kelly

**Affiliations:** 1 Department of Nursing and Midwifery, Sheffield Hallam University, Sheffield, UK; 2 Department of Allied Health Profession, Sheffield Hallam University, Sheffield, UK; 3 Department of Social Work, Social Care and Community Studies, Sheffield Hallam University, Sheffield, UK

**Keywords:** General practice nursing, Student nurses, Education, Recruitment, Survey, Questionnaire, Career intentions, Practice placements

## Abstract

**Background:**

The demand for General Practice services in the UK, and elsewhere, is rising quickly. In part, the increasing demand is from an aging population that requires management of multiple long-term conditions. The General Practice Nurse is increasingly taking on the role. It is acknowledged that if general practice is to be able to recruit sufficient General Practice Nurses (GPNs) to meet this increasing demand in the future, new graduate nurses must be encouraged to consider general practice as a viable career option. This research is part of a review of the Advanced Training Practice Scheme (ATPS) which supported clinical placements in participating general practices.

**Methods:**

The aim of the study was to examine nursing students’ perceptions of GP placements, and their effect upon career intentions following graduation from Sheffield Hallam University (SHU), in the UK. Interviews and an online survey were used collect data. Only the survey is reported here. The bespoke survey examined students’ views of: opportunities for learning new clinical skills and consolidating existing clinical skills; the learning environment in general practice and their views on a career in general practice.

**Results:**

One thousand one hundred twenty undergraduate adult-field nursing students were contacted, with a response rate of 41% (*N* = 462). Ninety respondents had a placement and, 92% (*N* = 84) viewed practice nursing positively, and 77% (*N* = 70) felt that the placement had transformed their views on general practice. The opportunity to participate in the management of the various aspects of chronic disease was identified by 84% (*N* = 76) of the students as a key new skill they had acquired. They also reported that they valued a team ethos, control over aspects of work, and the variety of health problems they encountered.

**Conclusion:**

The findings from this study demonstrate a positive experience arising from the provision of General Practice placements for nursing students. The use of ‘targeted’ placement schemes with appropriate support such as this may be seen as a viable way of exposing nursing students to General Practice nursing, and of encouraging new graduate nurses to consider General Practice nursing as a viable career option.

## Background

There is much reference in the international healthcare media to a mounting ‘workforce crisis’ in General Practice, and in the United Kingdom (UK), as with other post-industrialised countries, an inability to recruit and retain staff to work in General Practice is a significant problem [1–3]. At the same time, the UK government policy document *Five Year Forward View* [4] outlined large scale plans to move health care services from secondary care into the community. It is acknowledged that if general practice is to be able to meet this increasing demand on its services in the future, the necessary workforce resources must be put in place.

However, the demand for General Practice services in the UK, as elsewhere, is rising quickly, and it is now estimated that in the UK alone 340 million consultations are undertaken every year, an increase of 40 million over the last decade. The number of older people with multiple long-term conditions is also set to grow from 1.9 million in 2008 to 2.9 million by 2018, with the majority of these individuals being managed in primary care [5]. In UK general practice, most chronic disease management now comes under the aegis of General Practice Nurses (GPN). This has resulted in GPNs taking on a much greater responsibility for the surveillance and management of key long-term conditions (LTCs) such as asthma and diabetes [6, 7]. However, in the UK a 2015 report [8] found that 33.4% of GPNs surveyed are due to retire by 2020, and that 43.1% did not feel that their team currently had sufficient numbers of appropriately qualified and trained staff to meet the needs of their patients. This situation is also to be found in many other developed countries such as Australia and Canada (e.g. [3]).

### The emergent GPN workforce ‘crisis’

It should be noted here that in the UK General Practitioners (GPs) are independent small businesses. They are effectively sub-contracted by the National Health Service (NHS) in the UK to provide primary care services for the communities that they serve. As such they have a vested interest in only recruiting skilled and experienced GPNs as this removes the need for expensive and time-consuming education and training [9]. However, in spite of this, the Royal College of General Practice (RCGP) *Roadmap to Excellence* report [10] clearly articulated the need to attract more newly graduated nurses into General Practice if the predicted increases in workload and complexity of care are to be satisfactorily addressed. There is however still a perceived focus within nurse education on acute care as the clinical environment in which the majority of healthcare takes place. The result is a preference for acute care employment as the best way for new graduate nurses to consolidate their learning after graduation and before even considering a career in general practice. In addition, new graduate nurses are often under the (mis)impression that they ‘need’ to have some previous relevant clinical experience (either secondary care or community care) before applying for a GPN post [9]. Over time, this means that general practice has become regarded as something of a clinical ‘backwater’, an ‘easy ride’ suitable for older nurses with children, and one that is not immediately attractive to young, proactive newly graduated nurses [11–15]. The reasons for these attitudes amongst students are multifactorial, however career intentions are clearly influenced by these stereotypes [8, 11].

### Placement learning

There is a growing body of literature looking at the impact of placement learning on students’ experiences. The generic literature on the topic is significant and established (e.g. [16, 17]) and not for discussion here, however a growing number of studies and evaluations (e.g. [18–22] and [23–30]) have looked specifically at the influence of general practice placements. These studies identified the importance of factors such as the provision of 1:1 support during the placement experience. The prevailing culture, perceived workload and opportunities for learning are also implicated in the quality of the placement experience. The ability to develop a sense of ‘belonging’ to a team, to be able to place problems in their context and to rehearse future roles are also seen as important by students. The findings from these various international studies (from the UK and Australia in particular) indicate a generally positive placement experience, and although the placements did provide some impetus for consideration of a career in general practice, some of the evidence is confusing and contradictory (e.g. [31, 32]), and little is currently known regarding the true impact of primary care placements upon students’ career intentions.

Historically in the UK primary care placements have been based upon community nursing placements [21, 24] working with teams of nurses who typically see patients in their own homes. The students described this type of primary care as ‘real’ nursing and valued the skills and independence that the role allowed. In contrast, nursing students have only tended to visit general practices for short periods of time, as part of these community placements. During these visits, they spend the day observing, by ‘sitting in’ with a GP during their surgery rather than working with a GPN [9].

This situation, allied to a healthcare curriculum and an educational ‘system’ that is still biased towards secondary care, has resulted in student nurses having a chronic lack of understanding of how twenty first century General Practice ‘works’. In addition, students in the UK and elsewhere have had little, if any practical experience of the GPN role and what it currently involves (e.g. [9]). The most recent UK survey [8] found that only 27% of GPs currently offered placements for undergraduate nursing students, compared to 61.5% offering placements to undergraduate medical students. Consequently, there has been no real opportunity for students to positively experience General Practice, and all the evidence points to a lack of knowledge and understanding of the role of the GPN and all that it has to offer.

### Addressing the problem

There have been a number of proposed solutions to address the GPN recruitment crisis in the UK and elsewhere. In a number of areas within the UK, GPs are now being commissioned to provide targeted placements for student nurses to provide opportunities to gain an in-depth and sustained exposure to, and experience of, General Practice nursing. The idea is that this exposure to General Practice (GP) placements will encourage students to consider applying for a GPN post upon graduation [1, 5, 9]. These placement schemes are funded by Health Education England (HEE) and have been variously known as ‘Community Education Provider Networks’ (CPENs) or ‘Advanced Training Practice Schemes’ (ATPS) and are part of the UK National Training Hubs Initiative (NHTI) [29].

The scheme being studied covers a large geographical area in the north of England [29]. The area is predominantly urban in nature and has a population with a lower than average socio-economic status and poorer health profile [33]. To date, there have been several evaluations of various GP placement schemes in the UK (e.g. [5, 9, 23, 29, 32]) demonstrating varying degrees of success. These studies have tended to focus upon evaluation feedback from students and nurse mentors rather than on the quality of the overall educational experience. On the basis that a positive placement experience helps to challenge students’ perceptions of where they wish to work on graduation, increasing our understanding of what constitutes a positive learning experience in general practice is vital.

#### Aims and objectives

The overarching study aim was to examine the influence of GP placements on students’ career intentions following graduation. The specific objectives were:

1. To examine whether a General Practice placement was positively associated with an interest in General Practice Nursing as a career option
2. To examine whether experiencing a placement in General Practice changed students’ view of General Practice as a career option
3. To examine the students’ perceptions of the key skills that they learned whilst on placement

## Methods

An online survey was used to elicit students’ views on their clinical placements in general practice. Participants were all undergraduate adult nursing students at Sheffield Hallam University (SHU) in the UK who started training between 2014 and 2017 (years 1 through 3).

### Recruitment

All adult field students spend a total of 17 weeks on placement each year, and gain experience in a variety of different clinical settings, but hospital wards still predominate the placement ‘circuit’. At SHU only the ‘adult field’ students currently have placements in general practice. Participants were provided with detailed information about the study through an announcement on the course website. Following the announcement, an individualised email invitation to participate in the study, containing an electronic link to the online survey, was sent to all ‘adult field’ students. In addition, targeted reminders were sent to non-responders after 2 and 4 weeks. Participants’ completion of the online survey via their personalised email (which contained a detailed information sheet and contact details for the study) was taken as the provision of informed consent. An ‘iPad mini’ was offered via a prize draw as an incentive to participate. Although the emails were individualised, no identifiable data was collected or retained by the survey (e.g. student names).

### Data collection

The content of the survey was based upon expert opinion (derived from experienced ‘link’ clinicians from partner organisations), the findings from a rapid literature review and a ‘hand search’ of the ‘grey’ literature. There were a number of papers (e.g. [18]) that were identified as being particularly relevant. Following participant feedback from a small pilot survey relating to readability (*n* = 6 students), the final online survey was emailed to the students using Survey Monkey© software.

The survey began with an initial screening section which confirmed the student was a registered for a nursing degree at SHU and collected basic demographic details, including *age group, gender* and *year of study*. Students were then asked if they had experienced a placement in general practice. For those students who had not had a general practice placement the survey ended there, with a note thanking them for their participation thus far.

Those students who had experienced a placement in general practice were then asked to continue the survey and answer a series of further questions relating to their experiences. These questions asked the students about (a) the opportunities for learning new clinical skills, (b) the opportunities to consolidate existing clinical skills, (c) the learning environment in general practice and (d) their views on a career in general practice.

The list of clinical skills was derived from the students’ practice placement booklets, which provides a definitive list of key skills for students. Clearly some of the clinical skills were not suitable for general practice and the expert panel provided a consensus list of skills available in this clinical environment. We also provided a free text box for any other activities not listed. The students were asked to tick each skill that they had practiced on placement, from a list. For the purposes of the paper, the skills were then further sub-divided into ‘technical’ clinical skills and ‘non-technical’ skills. See Tables 2 and 4 for the content of the questions.

### Data analysis

The online survey data was downloaded, cleaned and formatted and response frequencies were calculated for each question. Statistical significance was set at *p* < 0.05. To ascertain whether there was any significant difference in the groups’ attitudes to, and perceptions of, general practice, univariate analysis (χ^2^ statistical test) was used to test for differences between those who had a general practice placement and those who had not. Subsequent descriptive statistical analysis was used for the students who had experienced a general practice placement. All analyses were conducted using SPSS v22 [34].

### Ethical approval for the study

Ethical approval was obtained from the SHU Faculty Research Ethics Committee (Ref: H447), and research governance protocols were adhered to throughout the study. All data was anonymised by the removal of any identifiable information, to maintain confidentiality and to ensure that no individual could be recognised in any subsequent report or publication. Given that this was a university-based study involving students, great care was taken to avoid any perception of coercion. Particular emphasis was given to reassuring the students that (a) they had the right to refuse to take part and (b) they would not be disadvantaged if they chose not to take part.

## Results

In total, 460 participants completed the screening part of the questionnaire. The total population of students registered in undergraduate nursing eligible to participate was 1120 giving a response rate of 41%, which is in line with expectations for this type of online survey.

Table 1 below shows some basic demographic information for the participants. The age and gender profile is roughly comparable to other nursing courses, and therefore is assumed to be a representative sample. The high percentage of age 30+ students is indicative of the significant number of ‘mature’ students that undertake their nurse training at the University. The Chi Squared test found no significant difference in the characteristics between those students with a GP placement and those without one.

**Table 1:** Student demographic information

	GP placement		*x*^2^test
	No: N (%)	Yes: N (%)	
	370 (80.5%)	90 (19.5%)	
Age range			
• 18–20	92 (24.9)	26 (28.9)	0.210
• 21–23	103 (27.8)	23 (25.6)	
• 24–29	90 (24.3)	14 (15.6)	
• 30+	85 (23.0)	27 (30.0)	
Gender			
• Female	350 (94.6)	87 (96.7)	0.419
• Male	20 (5.4%)	3 (2.3%)	

From a statistical point of view, comparing differences purely by intake cohort was considered to be of little value, since the March cohorts tend to be much smaller than the September cohorts, and the March 17 cohort had not yet had any opportunity to experience general practice. It was therefore decided to *aggregate* the total number of students who had experienced a GP placement. The remainder of the findings therefore focused upon the students (*n* = 90) who had experienced a placement in general practice. (see Table 2).

**Table 2:** Skills maintenance and acquisition on placement

Questions	N (%)
Which *existing* skills were you able to practice on placement?	
Technical skills (top 5 answers):	
1) Injection technique(s)	71 (79)
2) Manual blood pressure (BP) measurement	69 (77)
3) Wound care/assessment/dressing	69 (77)
4) Holistic 1:1 patient care	65 (72)
5) Administration of medication	65 (72)
Non-technical skills (top 5 answers):	
1) Increased understanding of long-term conditions (LTCs)	76 (84)
2) Patient assessment	71(79)
3) Health promotion/education advice	71(79)
4) Patient consultation	69 (77)
5) Communication	69 (77)
Which *new* skills did you develop?	
1) Management of LTCs e.g. diabetes	76 (84)
2) Patient assessment	76 (84)
3) Health promotion/education advice relating to managing LTCs	71 (79)
4) Consultation skills	69 (77)
5) Increased knowledge of different medications relating to LTCs	64 (71)

Table 2 lists the top five most cited skills in the clinical and non-clinical groupings. The students were also provided with a free text box and asked to identify which new skills they were able to learn and practice in general practice. Management of long-term conditions featured strongly in these new skills.

In Table 3, the students were asked to select from statements about the impact of the placements upon their views of general practice, and what effect it would have upon their career intentions. A cross tabulation of these responses found that 78% of them responded that the placement had positively influenced their career intentions.

**Table 3:** The impact of the general practice placement upon students’ career intentions

Question:	Yes: N (%)	No: N (%)	Not sure: N (%)
Would you *seriously* consider a career in general practice, once qualified?	43 (47.8)	14 (15.6)	33 (36.7)
Did the placement alter your views about a career in practice nursing?			
• Yes (positively)			70 (77.7)
• Yes (negatively)			5 (5.6)
• No (already positive)			14 (15.6)
• No (already negative)			1 (1.1)

In Table 4, the students who had responded ‘yes’ (positively) to the previous question (see Table 3) were then asked to provide a more detailed breakdown of their answer. To provide a narrative of their responses, the students’ top 5 responses were grouped under three separate headings; relating to (a) the general practice working environment, (b) perspectives on the GPN role and (c) the overall placement experience being offered.

**Table 4:** Factors that positively altered the students’ perceptions of general practice

GP Placement students	N (%)
If you answered ‘yes’ (positively) to the question in Table 3, what altered your views in a positive way?	
The working environment (top 5 answers):	
• Regular working hours	67 (74.4)
• Being part of a friendly, welcoming team	66 (73.3)
• ‘Family-friendly’ environment	66 (73.3)
• Learning about the role(s) of other professions (e.g. GP)	60 (66.7)
• Working collaboratively within a friendly team	55 (61.1)
The GPN role (top 5 answers):	
• A better understanding of the general practice nurse (GPN) role	74 (82.2)
• Long term conditions management	67 (74.4)
• Seeing the GPN working autonomously	59 (65.6)
• Having control over your own workload	58 (64.4)
• Having time for 1:1 patient care	62 (68.9)
The overall student experience ‘package’ (top 5 answers):	
• 1:1 time with mentor	69 (76.7)
• Good variety of patients	68 (75.6)
• Improving technical skills	66 (73.3)
• Good variety of skills opportunities	62 (68.9)
• Improving non-technical skills	62 (68.9)

## Discussion

The findings from this study indicate that when students are exposed in a focused, supported manner to general practice, they view the placement experience extremely positively. In this study, 92% of the students with a placement in General Practice viewed practice nursing positively, and 77% felt that the placement had positively transformed their views on general practice. In addition, the opportunity to fully engage in the management of chronic disease was identified by 84% of the students as a key new skill they had acquired. They also valued a team ethos, control over aspects of the GPN workload, and the variety of health problems they encountered.

As with this study, a number of other studies (e.g. [5, 9, 21, 23]) found a positive correlation between a good placement experience and students’ career intentions. There are a number of factors that students identify as important to a positive placement experience [33, 34]. The first of these relates to the working environment and their perceptions of how they are treated. The friendly nature of the general practice environment, together with one-to-one mentorship and exposure to a variety of learning experiences were all seen as positive enablers for the students. A significant percentage of the students (73%) in this study reported being treated as part of a friendly welcoming team, and that a small, friendly team combined with family-friendly working (73%) presented general practice as an attractive career choice which corresponds with our previously published qualitative findings [9, 23] and the findings from both McInnes et al. [25, 26] and Peters et al’s [28] studies. In addition, a greater understanding of team roles and the way in which the general practice team worked together collaboratively were also highlighted as important by students. These findings are consistent with the findings from a number of other international studies [35, 36] and the evidence from a number of recently published integrative reviews from Australia [33, 34, 37].

One of the significant aspects of the study examined the students’ views on the opportunities to practise clinical skills. Approximately 76% of the students highlighted the wide variety of patients and conditions, together with the opportunity and significantly the time to practise a variety of key *technical* and *non-technical* clinical skills. The opportunity to fully participate in patient care in a meaningful and holistic way was also afforded by the amount of time that the students were able to spend on a 1:1 basis with their mentors. The ability to develop a relationship with their mentor was highlighted by 77% of the students as a positive attribute of the general practice placement. This corresponds with a number of study findings (e.g. [25–28]) that the level of student support from staff in general practice was seen as a positive aspect of the placement. In addition, the expansion of the GPN role into the management of long-term conditions meant that the opportunity to participate in the management of chronic disease [38] was identified by 84% of the students as an important new skill they had acquired whilst on their placement. The opportunity to learn about and participate in 1:1 patient consultations and the holistic management of chronic conditions such as diabetes outside of the hospital setting were also highly valued by the students [9, 23].

Linked to this, the qualitative component of this research project [9, 23] also identified an increased understanding and appreciation of the widened scope of the GPN role emerged as an important influence, particularly in altering students’ views in a positive way. Approximately 80% of the students surveyed identified that a better understanding of the role of the GPN was central to their decision. And, in the interviews, the fact that the GPNs were now seen to have a significant degree of autonomy in their role, and that they were able to exert and maintain a degree of control over their workload and have the time to deliver holistic, patient-centred care were all perceived as significant, positive factors [9, 23].

In particular, the level of autonomy afforded to GPNs in the management of individuals with chronic diseases [35] and long-term conditions was highlighted as significant by approximately two thirds of the students [23]. These findings would appear to contradict the findings from for example Chowthi-Williams [30] in the UK and, McInnes et al. [25, 26] and McKenna et al. [27] in Australia that the GPN role was rather limited and of low acuity. Indeed, the participants in McKenna et al’s study concluded that little independent action or critical thinking would be required to carry out the role. It may be argued that the role of the GPN in the UK and Australia has developed beyond recognition over the last 5 years or so and that this may be attributed to a combination of the shortage of GPs, the increased demands upon general practice and to the importance being placed upon chronic disease management in primary care [38, 39].

However, this is contradicted by the 2015 and 2017 papers published by Bloomfield et al. [31, 32] which both reported that, in spite of the majority of Australian students in their study having experienced a primary care placement, the number of students identifying that they would consider a career in general practice was small. The study compared tertiary care, secondary care and primary care as potential career options. The authors found that primary care was not seriously regarded as a viable career option by the majority of the students that were surveyed. Crucially, they also found no association between students’ placement experiences and their career intentions in general practice. Larsen et al. [40] also found that participants did not see themselves working in general practice until later on in their career trajectories. As McKenna and Brooks [41] note, students often have a preconceived idea about where they want to work upon graduation and much of the evidence (e.g. [31, 32, 35, 40, 41]) would suggest that neophyte adult field students still gravitate towards secondary care areas such as intensive care, critical care and emergency care. But what we don’t know is what was the nature and quality of the primary care experience that they had on their placements.

There are a number of wider issues that have contributed to the current situation. The importance of a good working partnership between education providers and the clinical environment cannot be overstated [10, 24, 25], and this is particularly true of general practice [9]. The geographical and social isolation of a small team, with a small number of mentors requires a significant commitment from the HEI in terms of the practical support required to sustain the placements. Historically nurse education has subscribed to a version of the ‘repair and remediate’ medical model of illness and has yet to fully engage with the primary care ‘public wellness’ agenda [42–45]. In addition, secondary and tertiary care facilities are able to accommodate large numbers of student placements in a single setting. This arrangement has provided nurse education providers with a cost-effective way of meeting their commitments for student placements and has provided hospitals with a ready supply of newly qualified nurses [42–45]. HEIs have been slow (and reluctant) therefore to acknowledge and address the inexorable move in focus from secondary to primary care [42–44], both in terms of the students’ placement experience and the undergraduate nursing curriculum [46, 47]. This reluctance may have had a negative impact upon the ability and confidence of newly qualified nurses to work in primary care on graduation [39–41]. It may also have contributed to a number of negative preconceptions regarding general practice nursing [33, 34, 39–41].

Many of the previous studies in the UK (e.g. [5, 21, 30]) have been criticised for the small numbers of students involved. However, SHU is one of the largest providers of nurse education in the UK, and as such has access to a substantial placement circuit covering a large geographical area. As an organisation, SHU is used to dealing with large numbers of students, and the need to develop sustainable new placements for students in primary care has, in part, supported (and driven) the development of the ATPS scheme. As a result, this study has had access to larger numbers of students who have had general practice placements than in previous studies. It is also the quantitative part of a larger project, the qualitative components of which have already been reported upon [9, 23]. Given all the findings from this study, further expansion of the ATPS model providing increased access to general practice placements may serve to positively influence the views of these students. The findings from this study and the companion qualitative papers [9, 23] clearly demonstrate the importance of the ATPS placements in general practice in positively influencing students’ career intentions, and positively influencing the attitudes of the GPs towards them.

### Limitations to the study

There are a number of acknowledged limitations to this study. In terms of generalisability, the student participants were all recruited from one single university in the UK, and the online survey was a self-report measure. Although the response rate of 41% is in line with expectations for this type of survey, it is by necessity self-selecting and may therefore not accurately represent the views of all the students.

Finally, the survey used an investigator developed questionnaire, rather than an existing, validated tool. None of the existing tools were deemed suitable since they did not accurately reflect the issues being addressed. In addition, the survey was taken at one single point in the students’ educational trajectory (which could have been at any time over the 3 years of the programme), and repetition of the survey in a time series may give a more robust perspective on the views of the students as they progress through their course.

## Conclusions

The requirement to address the longstanding recruitment issues inherent in general practice nursing has driven the development of initiatives such as the one being evaluated here. If there is no clear recruitment and retention strategy put in place to increase the numbers of GPNs to both replace those GPNs due to retire within the next 5 years, then there is a ‘perfect storm’ brewing in which there will be an acute shortage of GPNs at a time when the workload in primary care is predicted to be at its greatest. The fact that approximately two thirds of the students surveyed in this study changed their views positively after the placement may be viewed as a vindication of the ATPS scheme as part of a long-term strategy to positively influence students’ careers intentions. This study, and others, is part of growing body of evidence that targeted, well-supported general practice placements are an effective way to positively influence students’ career intentions. The overarching philosophy of the ATPS is to promote s*ustainable* cultural change in general practice nursing by widening student nurse access to general practice placements. Through the ATPS, GPs are then supported to ‘grown their own’ GPNs by recruiting new graduate nurses. As the ATPS becomes fully embedded, the idea of ‘growing your own’ is beginning to change the prevailing culture within general practice.

## Data Availability

We would have to seek permission from the funder (Health Education Yorkshire & Humber) to make the data available. The datasets used and analysed during the study are available from the corresponding author upon reasonable request.

## Abbreviations

ATPS: Advanced Training Practice Scheme
CPEN: Community provider education network
GP: General Practitioner
GPN: General practice nurse
HEE: Health Education England
HEI: Higher education provider
LTC: Long term condition
NHS: National Health Service
NHTI: National training hubs initiative
QNI: Queen’s Nursing Institute
SHU: Sheffield Hallam University
UK: United Kingdom

## References

[1] Lewis R, Kelly S (2017). Would growing our own practice nurses solve the workforce crisis?. Pract Nurs.

[2] Australian Primary Healthcare Nurses Association (2017). APNA annual report 2017.

[3] Freund T, Everett C, Griffiths P, Hudon C, Naccarella L, Laurant M (2015). Skill mix, roles and remuneration in the primary care workforce: who are the healthcare professionals in the primary care teams across the world?. Int J Nurs Stud.

[4] NHS England. Five-year forward view: NHS England; 2014. https://www.england.nhs.uk/wp-content/uploads/2014/10/5yfv-web.pdf. Accessed 27 Dec 2018

[5] McLaren WK, Quinlivan L (2016). Developing student nurse placements in general practice. Gen Pract Nurs.

[6] Ball J, Maben J, Griffiths P (2015). Practice nursing: what do we know?. Br J Gen Pract.

[7] Griffiths P, Maben J, Murrells T (2011). Organisational quality, nurse staffing and the quality of chronic disease management in primary care: observational study using routinely collected data. Int J Nurs Stud.

[8] Queen’s Nursing Institute (QNI) (2015). General practice nursing in the 21st century: a time of opportunity.

[9] Lewis R, Kelly S (2018). GP perspectives on clinical placements for student nurses in general practice: can a community of practice help to change the culture within general practice?. BMC Fam Pract.

[10] Royal College of General Practitioners. Nursing in primary care – ‘a roadmap to excellence’: Royal College of General Practitioners; 2014. www.rcgp.org.uk/membership/.../C3DC3C7A6FA344768E7319D66489008B.ashx. Accessed 21 Mar 2018

[11] Ashley C, Halcomb E, Brown A (2016). Transitioning from acute to primary health care nursing: an integrative review of the literature. J Clin Nurs.

[12] Ashley C, Halcomb E, Peters K, Brown A (2017). Exploring why nurses transition from acute care to primary health care employment. Appl Nurs Res.

[13] Ashley C, Brown A, Halcomb E, Peters K (2018). Registered nurses transitioning from acute care to primary healthcare employment: a qualitative insight into nurses’ experiences. J Clin Nurs.

[14] Ashley C, Halcomb E, Brown A, Peters K (2018). Experiences of registered nurses transitioning from employment in acute care to primary health care-quantitative findings from a mixed-methods study. J Clin Nurs.

[15] Betony K (2012). Clinical practice placements in the community: a survey to determine if they reflect the shift in healthcare delivery from secondary to primary care settings. Nurse Educ Today.

[16] Levett-Jones T, Lathlean J, Higgins L, McMillan M (2008). The duration of clinical placements: a key influence on nursing students’ sense of belongingness. Aust J Adv Nurs.

[17] Levett-Jones T, Fahy K, Parsons K, Mitchell A (2006). Enhancing nursing students’ clinical placement experiences: a quality improvement project. Contemp Nurse.

[18] Baglin M, Rugg S (2010). Student nurses’ experiences of community-based practice placement learning: a qualitative exploration. Nurse Educ Pract.

[19] Bennett P, Jones D, Brown J, Barlow V (2013). Supporting rural/remote primary health care placement experiences increases undergraduate nurse confidence. Nurse Educ Today.

[20] Carryer J, Halcomb E, Davidson, Bos E, Alinaghizadeh H, Saarikoski M, Kaila P (2015). Factors associated with student learning processes in primary health care units: a questionnaire study. Nurse Educ Today.

[21] Gale J, Ooms A, Sharples K, Marks-Maran D (2015). The experiences of student nurses on placements with practice nurses: a pilot study. Nurse Educ Pract.

[22] Halcomb E, Patterson E, Davidson P (2012). Practice nurses experiences of mentoring undergraduate nursing students in Australian general practice. Nurse Educ Today.

[23] Lewis R, Kelly S (2018). Changing hearts and minds: examining student nurses’ experiences and perceptions of a general practice placement through a ‘community of practice’ lens. BMC Med Educ.

[24] Löfmark A, Hansebo G, Nilsson M, Törnkvist L (2008). Nursing students’ views on learning opportunities in primary health care. Nurs Stand.

[25] McInnes S, Peters K, Hardy J, Halcomb E (2015). Clinical placements in Australian general practice: the experience of pre-registration nursing students. Nurse Educ Pract.

[26] McInnes S, Peters K, Hardy J, Halcomb E (2015). Clinical placements in Australian general practice: the views of registered nurse mentors and pre-registration nursing students. Nurse Educ Pract.

[27] McKenna L, Parry A, Kirby C, Gilbert K, Griffiths R (2014). Learning in primary health care settings: Australian undergraduate nursing students’ perspectives. J Nurs Educ Pract.

[28] Peters K, McInnes S, Halcomb E (2015). Nursing students’ experiences of clinical placement in community settings: a qualitative study. Collegian.

[29] Lewis R, Kelly S, Berwick L (2017). An evaluation of the health education England working across Yorkshire & the Humber Advanced Training Practice Scheme (ATPS).

[30] Chowthi-Williams A, Harris D, Curzio J (2010). Evaluation of a primary care pre-registration programme in London. Pract Nurs.

[31] Bloomfield J, Gordon C, Williams A, Aggar C (2015). Nursing students’ intentions to enter primary care as a career option: Findings from a national survey. Collegian.

[32] Bloomfield JG, Aggar C, Thomas THT, Gordon CJ (2018). Factors associated with final year nursing students’ desire to work in the primary health care setting: findings from a national cross-sectional survey. Nurse Educ Today.

[33] Doncaster Health Profile. London: Public Health England; 2017. http://fingertipsreports.phe.org.uk/health-profiles/2016/e08000017.pdf&time_period=2016. Accessed 1 Feb 18.

[34] SPSS V22 statistical package 2018. IBM Industries USA.

[35] van Iersel M, Latour CHM, de Vos R, Kirschner PA, Scholte Op Reimer WJM (2018). Perceptions of community care and placement preferences in first-year nursing students: a multicentre, cross-sectional study. Nurse Educ Today.

[36] Mackey S, Kwok C, Anderson J, Hatcher D, Laver S, Dickson C (2018). Australian student nurse’s knowledge of and attitudes toward primary healthcare: a cross-sectional study. Nurse Educ Today.

[37] van Iersel M, Latour CHM, de Vos R, Kirschner PA, Scholte Op Reimer WJM (2016). Nursing students’ perceptions of community care and other areas of nursing practice – a review of the literature. Int J Nurs Stud.

[38] Halcomb EJ, Davidson PM, Salamonson Y, Ollerton R, Griffiths R (2008). Nurses in Australian general practice: implications for chronic disease management. J Clin Nurs.

[39] Wojnar DM, Whelan EM (2017). Preparing nursing students for enhanced roles in primary care: the current state of prelicensure and RN-to-BSN education. Nurs Outlook.

[40] Larsen L, Reif R (2012). Frauendienst Baccalaureate nursing students’ intention to choose a public health career. Public Health Nurs.

[41] McKenna L, Brooks I (2018). Graduate entry students’ early perceptions of their future nursing careers. Nurse Educ Pract.

[42] Keleher H, Parker R, Francis K (2010). Preparing nurses for primary healthcare futures: how well do Australian nursing courses perform?. Aust J Prim Health.

[43] Cooper S, Cant R, Browning M, Robinson E (2014). Preparing nursing students for the future: development and implementation of an Australian bachelor of nursing programme with a community health focus. Contemp Nurse.

[44] Parker R, Keleher H, Francis K, Abdulwadud O (2009). Practice nursing in Australia: a review of education and career pathways. BMC Nurs.

[45] Peters K, Halcomb E, McInnes S (2013). Clinical placements in general practice; relationships between practice nurses and tertiary institutions. Nurse Educ Pract.

[46] Calma K, Halcomb E, Stephens M (2019). The impact of curriculum on nursing students’ attitudes, perceptions and preparedness to work in primary health care: an integrative review. Nurse Educ Pract.

[47] Byfield Zachary, East Leah, Conway Jane (2019). An integrative literature review of pre-registration nursing students’ attitudes and perceptions towards primary healthcare. Collegian.

